# Zika Virus Surveillance at the Human–Animal Interface in West-Central Brazil, 2017–2018

**DOI:** 10.3390/v11121164

**Published:** 2019-12-16

**Authors:** Alex Pauvolid-Corrêa, Helver Gonçalves Dias, Laura Marina Siqueira Maia, Grasiela Porfírio, Thais Oliveira Morgado, Gilberto Sabino-Santos, Paula Helena Santa Rita, Wanessa Teixeira Gomes Barreto, Gabriel Carvalho de Macedo, Jaire Marinho Torres, Wesley Arruda Gimenes Nantes, Filipe Martins Santos, William Oliveira de Assis, Andreza Castro Rucco, Rafael Mamoru dos Santos Yui, João Bosco Vilela Campos, Renato Rodrigues Leandro e Silva, Raquel da Silva Ferreira, Nilvanei Aparecido da Silva Neves, Michell Charlles de Souza Costa, Leticia Ramos Martins, Emerson Marques de Souza, Michellen dos Santos Carvalho, Marina Gonçalves Lima, Fernanda de Cássia Gonçalves Alves, Luiz Humberto Guimarães Riquelme-Junior, Luan Luiz Batista Figueiró, Matheus Fernandes Gomes de Santana, Luiz Gustavo Rodrigues Oliveira Santos, Samara Serra Medeiros, Larissa Lopes Seino, Emily Hime Miranda, José Henrique Rezende Linhares, Vanessa de Oliveira Santos, Stephanie Almeida da Silva, Kelly Araújo Lúcio, Viviane Silva Gomes, Alexandre de Araújo Oliveira, Julia dos Santos Silva, William de Almeida Marques, Marcio Schafer Marques, José Junior França de Barros, Letícia Campos, Dinair Couto-Lima, Claudia Coutinho Netto, Christine Strüssmann, Nicholas Panella, Emily Hannon, Barbara Cristina de Macedo, Júlia Ramos de Almeida, Karen Ramos Ribeiro, Maria Carolina Barros de Castro, Larissa Pratta Campos, Ana Paula Rosa dos Santos, Isabelle Marino de Souza, Mateus de Assis Bianchini, Sandra Helena Ramiro Correa, Renato Ordones Baptista Luz, Ananda dos Santos Vieira, Luzia Maria de Oliveira Pinto, Elzinandes Azeredo, Luiz Tadeu Moraes Figueiredo, Jeronimo Augusto Fonseca Alencar, Sheila Maria Barbosa de Lima, Heitor Miraglia Herrera, Renata Dezengrini Shlessarenko, Flavia Barreto dos Santos, Ana Maria Bispo de Filippis, Stephanie Salyer, Joel Montgomery, Nicholas Komar

**Affiliations:** 1 Laboratório de Flavivírus, Instituto Oswaldo Cruz, Fiocruz, Rio de Janeiro 21040-900, Brazil; abispo@ioc.fiocruz.br; 2 Laboratório de Imunologia Viral, Instituto Oswaldo Cruz, Fiocruz, Rio de Janeiro 21040-900, Brazil; helvergd@gmail.com (H.G.D.); lmopnogueira@gmail.com (L.M.d.O.P.); naideazeredo@gmail.com (E.A.); flaviab@ioc.fiocruz.br (F.B.d.S.); 3 Laboratório de Virologia, Faculdade de Medicina, Universidade Federal de Mato Grosso (UFMT), Cuiabá 78060-900, Brazil; lauramsmaia@gmail.com (L.M.S.M.); rah.kell_@hotmail.com (R.d.S.F.); nilvanei@hotmail.com (N.A.d.S.N.); michellcharlles2021@gmail.com (M.C.d.S.C.); le_ramos@hotmail.com (L.R.M.); emersonesem@gmail.com (E.M.d.S.); michellengalex@gmail.com (M.d.S.C.); renatadezengrini@yahoo.com.br (R.D.S.); 4 Laboratório de Biologia Parasitária, Programa de Pós-Graduação em Ciências Ambientais e Sustentabilidade Agropecuária, Universidade Católica Dom Bosco (UCDB), Campo Grande 79117-010, Brazil; grasi_porfirio@hotmail.com (G.P.); carvalhodemacedo@gmail.com (G.C.d.M.); jairemarinho@hotmail.com (J.M.T.); wesley_anantes@hotmail.com (W.A.G.N.); filipemsantos@outlook.com (F.M.S.); william.oliveira.assis@gmail.com (W.O.d.A.); drezacastro.rucco@hotmail.com (A.C.R.); rafayui99@gmail.com (R.M.d.S.Y.); bjoaocampos@gmail.com (J.B.V.C.); renatorleandro@gmail.com (R.R.L.e.S.); herrera@ucdb.br (H.M.H.); 5 Hospital Veterinário, Universidade Federal de Mato Grosso (UFMT), Cuiabá 78060-900, Brazil; thaismorgado@gmail.com (T.O.M.); assisbianchini@gmail.com (M.d.A.B.); correasandrahelena@gmail.com (S.H.R.C.); renato.ordones@outlook.com (R.O.B.L.); ananda.asv@gmail.com (A.d.S.V.); 6 Centro de Pesquisa em Virologia, Faculdade de Medicina, Universidade de São Paulo (USP), Ribeirão Preto 14025-099, Brazil; sabinogsj@usp.br (G.S.-S.); marcioschafer@msn.com (M.S.M.); ltmfigue@fmrp.usp.br (L.T.M.F.); 7 Biotério, Universidade Católica Dom Bosco (UCDB), Campo Grande 79117-010, Brazil; paulabiovet@ucdb.br (P.H.S.R.); marina-lima94@outlook.com (M.G.L.); fdecassia.alves@gmail.com (F.d.C.G.A.); bioriquelme@gmail.com (L.H.G.R.-J.); llb.figueiro@gmail.com (L.L.B.F.); matheussantana_21@hotmail.com (M.F.G.d.S.); 8 Laboratório de Ecologia de Populações e do Movimento, Programa de Ecologia e Conservação, Universidade Federal de Mato Grosso do Sul (UFMS), Campo Grande 79070-900, Brazil; wanessatgbarreto@gmail.com (W.T.G.B.); gu_tapirus@hotmail.com (L.G.R.O.S.); samarabio2013@gmail.com (S.S.M.); larissalopesseino@hotmail.com (L.L.S.); 9 Laboratório de Tecnologia Virológica, Bio-Manguinhos, Fiocruz, Rio de Janeiro 21040-900, Brazil; emily@bio.fiocruz.br (E.H.M.); jose.henrique@bio.fiocruz.br (J.H.R.L.); kelly.araujo@bio.fiocruz.br (K.A.L.); viviane.gomes@bio.fiocruz.br (V.S.G.); smaria@bio.fiocruz.br (S.M.B.d.L.); 10 Laboratório de Diptera, Instituto Oswaldo Cruz, Fiocruz, Rio de Janeiro 21040-900, Brazil; alexandre.bio.br@gmail.com (A.d.A.O.); wmarques.bio@gmail.com (W.d.A.M.); jafalencar@gmail.com (J.A.F.A.); 11 Laboratório de Virologia Molecular, Instituto Oswaldo Cruz, Fiocruz, Rio de Janeiro 21040-900, Brazil; barros@ioc.fiocruz.br (J.J.F.d.B.); leticia_bonfim1998@hotmail.com (L.C.); 12 Laboratório de Transmissores de Hematozoários, Instituto Oswaldo Cruz, Fiocruz, Rio de Janeiro 21040-900, Brazil; dcouto@ioc.fiocruz.br; 13 Centro de Reabilitação de Animais Silvestres (CRAS), Campo Grande 79037-109, Brazil; ccoutinhonetto@gmail.com; 14 Faculdade de Medicina Veterinária, Universidade Federal de Mato Grosso (UFMT), Cuiabá 78060-900, Brazil; christrussmann@gmail.com (C.S.); juh.ramos97@gmail.com (J.R.d.A.); ramosrkaren@gmail.com (K.R.R.); larissapratta@gmail.com (L.P.C.); anapaula.santosrosa@hotmail.com (A.P.R.d.S.); isamarinodesouza@gmail.com (I.M.d.S.); 15 Laboratory of Arbovirus Ecology, Arboviral Diseases Branch, U.S. Centers for Disease Control and Prevention (CDC), Fort Collins, CO 80521, USA; nap4@cdc.gov (N.P.); hannon.emily.r@gmail.com (E.H.); 16 Faculdade de Medicina Veterinária da Universidade de Cuiabá (UNIC), Cuiabá 78065-900, Brazil; barbara_cm02macedo@hotmail.com; 17 Global Epidemiology, Laboratory, and Surveillance Branch, Division of Global Health Protection, Center for Global Health, CDC, Atlanta, GA 30333, USA; wig9@cdc.gov (S.S.); ztq9@cdc.gov (J.M.)

**Keywords:** Zika, enzootic cycle, Brazil, zoonotic, plaque reduction neutralization test (PRNT)

## Abstract

Zika virus (ZIKV) was first discovered in 1947 in Uganda but was not considered a public health threat until 2007 when it found to be the source of epidemic activity in Asia. Epidemic activity spread to Brazil in 2014 and continued to spread throughout the tropical and subtropical regions of the Americas. Despite ZIKV being zoonotic in origin, information about transmission, or even exposure of non-human vertebrates and mosquitoes to ZIKV in the Americas, is lacking. Accordingly, from February 2017 to March 2018, we sought evidence of sylvatic ZIKV transmission by sampling whole blood from approximately 2000 domestic and wild vertebrates of over 100 species in West-Central Brazil within the active human ZIKV transmission area. In addition, we collected over 24,300 mosquitoes of at least 17 genera and 62 species. We screened whole blood samples and mosquito pools for ZIKV RNA using pan-flavivirus primers in a real-time reverse-transcription polymerase chain reaction (RT-PCR) in a SYBR Green platform. Positives were confirmed using ZIKV-specific envelope gene real-time RT-PCR and nucleotide sequencing. Of the 2068 vertebrates tested, none were ZIKV positive. Of the 23,315 non-engorged mosquitoes consolidated into 1503 pools tested, 22 (1.5%) with full data available showed some degree of homology to insect-specific flaviviruses. To identify previous exposure to ZIKV, 1498 plasma samples representing 62 species of domestic and sylvatic vertebrates were tested for ZIKV-neutralizing antibodies by plaque reduction neutralization test (PRNT_90_). From these, 23 (1.5%) of seven species were seropositive for ZIKV and negative for dengue virus serotype 2, yellow fever virus, and West Nile virus, suggesting potential monotypic reaction for ZIKV. Results presented here suggest no active transmission of ZIKV in non-human vertebrate populations or in alternative vector candidates, but suggest that vertebrates around human populations have indeed been exposed to ZIKV in West-Central Brazil.

## 1. Introduction

Zika virus (ZIKV) was first discovered in 1947 in Uganda but was not considered a public health threat until 2007 when it found to be the source of epidemic activity in Asia, including the South Pacific and Indian Ocean islands [1,2]. Epidemic activity spread to Brazil in 2014 and continues to spread in tropical and subtropical regions of the Americas, where the urban mosquito and main suspected vector *Aedes aegypti* is abundant [3]. There are records of ZIKV isolation from various species of mosquitoes of the genus *Aedes* in Africa and Malaysia [4]. Limited ecologic data from endemic regions of Africa reveal that a variety of zoophilic mosquitoes may also be vectors [5]. At these sites, ZIKV may be transmitted in a sylvatic cycle involving non-human primates and mosquitoes with incidental human exposure [6]. In addition, serological studies have detected hemagglutination-inhibition antibodies to ZIKV in cattle, horses, goats, ducks, and bats from Indonesia, complement-fixation antibodies in rodents from Pakistan, and neutralizing antibodies in orangutans from Borneo [7,8,9]. Humans develop disease mainly after being bitten by an infectious mosquito, but sexual and congenital transmission have also been reported [10,11].

In Brazil, the ZIKV epidemic occurred concurrently with an unusual increase in cases of microcephaly, especially in the Northeast region of the country [3,12]. The association of epidemiological data with the detection of ZIKV in amniotic fluid, fetal brain tissue, and the ability to infect neuronal progenitor cells reinforces this relationship [13,14,15]. In addition, a congenital zika syndrome, characterized by other neurological disorders without microcephaly such as delayed neuropsychomotor development and ocular lesions, has been described in infants of infected mothers in different periods of pregnancy [14,16]. Cases of Guillain–Barré syndrome were also associated with ZIKV infection in Brazil [17].

Zoonotic transmission networks in the Americas have not yet been adequately studied [18,19]. Despite ZIKV being zoonotic in origin, there is scarce information about the potential amplifying hosts other than humans for ZIKV in the Americas, and the role they might play in the virus maintenance and transmission. The present study is part of an overarching multi-country project to investigate potential enzootic transmission cycles of ZIKV in endemic tropical ecosystems of South America, including Brazil, Colombia, and Peru.

In Brazil, evidence of zoonotic ZIKV infection has been detected mainly in non-human primates. ZIKV RNA and anti-ZIKV antibodies have been detected in marmosets and capuchin monkeys, mostly from the Northeast region of the country [20,21]. Most recently, ZIKV RNA was also detected in carcasses of non-human primates during an epizootic outbreak of yellow fever in southeast Brazil, indicating exposure of non-human primates to ZIKV in Brazil [22]. A sylvatic maintenance cycle of ZIKV could not only precludes its control, but also create sylvatic zones of infection resulting in reemergence and potential outbreaks [19].

To further our understanding of the vertebrate host range for ZIKV in Brazil, we assessed the exposure of ZIKV among vertebrate species, including domestic and wild animals, such as amphibians, reptiles, birds and mammals, in regions where ZIKV was actively circulating in the country. We also collected a variety of mosquito species to have a better understanding of the vector roles of mosquitoes in tropical ecosystems.

The West-Central region was chosen based on historical records of reoccurring arbovirus epidemics and current reports of ZIKV transmission in the human population. The West-Central region reportted the highest incidence of ZIKV cases in 2017, with 39 cases/100,000 residents [23]. The state of Mato Grosso (MT) presented Brazil’s highest incidence not only in 2017 (63 cases/100,000 residents) but also in 2018 with 16 cases/100,000 residents [24].

Thus, the main objective of the present study is to obtain evidence of the zoonotic circulation of ZIKV and its potential amplifying hosts in Brazil by performing active surveillance for ZIKV in both wild and domestic animals, and in vector populations in close proximity to active human transmission areas. Our observations will lead to a better understanding of ZIKV’s ability to establish a sylvatic cycle outside of the urban transmission cycle, and its potential for zoonotic transmission or spillover into animal species.

## 2. Material and Methods

### 2.1. Study Sites

We conducted active ZIKV surveillance in vertebrate and mosquito species at two field sites of the West-Central region of Brazil. Field sites encompassed the metropolitan area of MT, including the cities of Cuiabá (15°35′56″ S 56°5′42″ W) and Várzea Grande, and the metropolitan area of the state of Mato Grosso do Sul (MS), including the city of Campo Grande (20°26′37″ S 54°38′52″ W), both metropolitan areas with estimated populations of over 895,000 residents each in 2019 [25]. A few field sites outside the metropolitan areas were also included.

Multiple subsites, mostly in metropolitan areas, were utilized in order to maximize the diversity of specimens collected and take advantage of locations where certain species were concentrated. We collected samples during four campaigns of around 2–3 weeks each, including two samplings during the dry season (April to May, and July to August 2017) and two samplings during the rainy season (October to November 2017 and February to March 2018).

### 2.2. Mosquito Sampling

Mosquito sampling targeted abundant peri-urban species, including *Aedes aegypti*, *Culex quinquefasciatus* and sylvatic species such as *Psorophora* spp. and *Haemagogus* spp. Mosquito collection occurred monthly, with supplemental collections made during the field campaigns. When possible, mosquito traps were co-located in space and time with vertebrate traps (within 500 m, and during the same two-week sampling period) so that data from vertebrate sampling could be linked with data from mosquito collections. We used multiple mosquito trap types deployed in several microhabitats to collect diverse diurnal and nocturnal mosquito species in microhabitats where humans, domestic, and wild animals occupy and rest during the night. Trap types used included per each field site: (1) two backpack aspirators or hand-held Insectazooka or Prokopak aspirators, (2) ten CDC light traps, sometimes baited with CO2, (3) eight BG-Sentinel Traps™ baited with human lure, octanol or CO2, and (4) fifteen resting traps for engorged mosquitoes.

Mosquito collections segregated by subsite, trap type, and date, were transported to the laboratory and kept frozen for further identification. Mosquitoes were identified and sorted by species, sex, and blood engorgement status using a dissecting scope and dichotomous keys [26,27]. Non-engorged mosquitoes were pooled by species, sex, subsite, and date of collection in cryovials. Pools included up to 25 specimens. Mosquito pools were homogenized in a mixer mill using sterile glass grinding beads and 800 microliters of grinding buffer (199 medium with antibiotics and antimycotics). After clarification by centrifugation, a 140 uL aliquot of the supernatant was removed for RNA extraction. To avoid virus detection from viremic blood meals rather than from mosquito salivary glands and bodies, engorged mosquitoes were separated individually for further analysis.

### 2.3. Vertebrate Sampling

Because prevalence data does not currently exist for ZIKV in this region for our target species, we aimed to sample at least 30 individuals per vertebrate species, per fieldwork campaign.

Collections targeted abundant domestic species including horses, cattle, sheep, dogs, cats, and poultry, as well as peri-urban wildlife including opossums, coatis, capybaras, bats, small rodents, turtles, frogs, snakes, and non-human primates. Field teams sampling vertebrates were led by veterinarians and biologists.

We used a variety of vertebrate trap types and site microhabitats to collect diverse wildlife species. Sampling locations included urban parks, zoo, campuses of local universities, state police equine facilities and equestrian societies, residential neighborhoods, zoonosis control centers, veterinary hospitals, shelters, ranches, nature reserves, and a wild animal rescue center.

Six standard traps were used to capture free-ranging flying and terrestrial wild animals in forested areas of MT and MS. Larger-size baited walk-in traps or anesthetic darting were used for free-roaming coatis and capybaras, and turtle traps were used for aquatic reptiles in MS. Mist nets were used to capture bats, Tomahawk and Sherman live traps were deployed for medium and small ground-dwelling mammals, respectively. Nocturnal anurans were manually captured. Animals were identified, weighed, measured, blood-sampled, and when practical, marked or tagged before release.

Wild animals were transported within their closed and covered traps by hand to a centralized processing station located within the collection subsite. We collected whole blood in tubes containing buffered sodium citrate solution by venipuncture. When enough volume was available, whole blood samples were centrifuged and plasma removed for antibody detection.

Domestic animals were handled and blood sampled at the site of domestic animal enclosures, or homes (for pet dogs and cats), and subsequently returned to their enclosures.

### 2.4. Data Collection and Analysis

We collected all field data via handheld Android devices and a Commcare application (Dimagi, Cambridge, MA, USA) that directly uploaded all data into an electronic, cloud-hosted database. We used electronic barcodes to identify any samples collected in the field and link to the laboratory results. Geographic positioning system (GPS) coordinates and environmental variables were collected at each sampling location and linked to the laboratory results.

### 2.5. Laboratory Testing

#### 2.5.1. Real-Time Reverse Transcriptase Polymerase Chain Reaction (RT-PCR) for Flaviviruses

A SYBR Green real-time RT-PCR method for the universal detection of flaviviruses was used to test all whole blood and mosquito samples for flaviviral nucleic acid at Laboratório de Flavivírus (LABFLA) and Imunologia Viral (LIV) of Instituto Oswaldo Cruz (Fiocruz, Rio de Janeiro, Brazil). First, RNA was extracted from whole blood samples and triturated mosquitoes using the ZR-Viral RNA or DNA/RNA kits (Zymo Research, Irvine, CA, USA) according to the manufacturer’s instructions. RNA samples were then tested for flaviviruses by a genus-specific real-time RT-PCR, based on the amplification of a 269–272 nucleotide region at the N terminus of the NS5 gene, as previously described [28]. The above-mentioned protocol was chosen as the screening method based on its desirable high sensitivity achieved by using degenerate primers. This protocol has experimentally demonstrated not only the capacity of detecting various flaviviruses that circulate in Brazil [29], as well as ZIKV in various dilutions, but was also capable of detecting novel flaviviruses [30].

#### 2.5.2. Real-Time RT-PCR for ZIKV and Sanger Nucleotide Sequencing

Samples that presented an amplicon melting curve at a temperature above 75°C by the SYBR Green real-time RT-PCR for flaviviruses were considered positive and then selected for a specific TaqMan^®^ real-time RT-PCR targeting the envelope gene of ZIKV [1], followed by nucleotide sequencing. Briefly, amplicons were purified using a commercial kit (QIAGEN, Hilden, Germany) and subjected to sequencing reaction with forward and reverse primers in separate reactions using a commercial kit (Applied Biosystems^®^, Foster City, CA, USA). Sequences were determined at a Fiocruz nucleotide sequencing center using an ABI 3730 DNA Analyzer (Applied Biosystems^®^, Foster City, CA, USA). Analysis was performed using Bioedit (http://www.mbio.ncsu.edu/bioedit/bioedit.html) and Mega-6 (https://www.megasoftware.net), and the sequences’ identity was obtained by nucleotide BLAST (http://blast.ncbi.nlm.nih.gov/Blast.cgi).

#### 2.5.3. Plaque Reduction Neutralization Test (PRNT_90_)

All plasma samples were heat-inactivated and tested by the 90% plaque-reduction neutralization test (PRNT_90_) for their ability to neutralize plaque formation by referencing ZIKV following standard protocols [31]. Samples with neutralizing antibodies for ZIKV were also submitted to PRNT_90_ for referencing yellow fever virus (YFV), West Nile virus (WNV) and dengue virus serotype 2 (DENV-2). Reference viruses were provided by LABFLA and Laboratório de Tecnologia Virológica (LATEV) of Fiocruz, from their arbovirus stocks. Reference viruses used for PRNT_90_ were previously tested by real-time RT-PCR for Chikungunya virus (CHIKV), DENV, YFV, WNV, and ZIKV, followed by partial nucleotide sequencing of the N terminal region of NS5 gene to confirm viral identity and discard viral contamination. High identity scores were obtained with the following sequences deposited at GenBank: ZIKV (KX197205), DENV-2 (JX669478), YFV (DQ100292), and WNV (KR348966). Fourth-passage preparation of ZIKV strain ES2916/2015 (isolated in the Espírito Santo state, Brazil, in September 2015) was used in this study. Serum samples of experimentally infected rhesus macaques (*Macaca mulatta*) and positive human samples were used as positive controls. Diluent media and plasma samples of wild animals that had PRNT_90_ titers <10, were used as negative controls.

Briefly, in a biosafety level three facility (BSL3) of Fiocruz, plasma samples were initially aliquoted and inactivated in a 56 °C water bath for 30 min. Inactivated aliquots were then initially screened at a dilution of 1:10 and those that neutralized ZIKV by at least 90% were further tested at serial two-fold dilutions to determine 90% endpoint titers. Plasma samples were considered having ZIKV-neutralizing antibodies when a plasma dilution of at least 1:20 reduced no less than 90% of the formation of ZIKV viral plaques. Plasma samples with ZIKV-neutralizing antibodies in monotypic reactions that failed to neutralize 90% of virus plaques of each of DENV-2, WNV and YFV in Vero cells were considered seropositive for ZIKV. In PRNT, positive reactions are considered monotypic when a sample neutralizes only one of several viruses challenged. Considering monotypic reactions to be the most reliable indicator of previous ZIKV infection, plasma samples that were ZIKV-seropositive and had PRNT_90_ titers ≥ 10 for any of the three other flaviviruses tested were considered heterotypic reactions indicating past infection(s) with undetermined flavivirus(es), as previously reported in serosurveys for other arbovirus groups [32]. Because of the low specificity of anti-flavivirus antibodies, plasma samples that presented PRNT_90_ titers for ZIKV of 10, in either monotypic or heterotypic reactions, were considered seronegative. To save resources, plasma samples with PRNT_90_ titers ≥10 for any of the three other flaviviruses were not further tested to determine endpoint titers.

### 2.6. Ethics Clearance

This project was approved by the U.S. Centers for Disease Control and Prevention Institutional Animal Care and Use Committee (Protocol number 2808SALMULX-A2-08/31/2016), and additional local institution approvals were obtained from each partner institution. This study was also approved by the Animal Ethics Committee of the Universidade Federal de Mato Grosso (UFMT-23108.169037-06/23/2016-24) and Universidade Católica Dom Bosco (UCDB-001-03/23/2017; 005-04/24/2017), in compliance with the requirements of Brazilian Law 11,794/2008, decree 6899/2009 and regulations issued by the National Council for Experimental Animals Control (CONCEA), which rules on the scientific use of animals, including the principles of the Brazilian Society of Science on laboratory animals.

Wildlife capture and sampling were authorized by Brazilian environment state agencies from MT (SEMA 201624/2017) and MS (IMASUL 61405959/2016) for animal collections in public parks, as well as the Instituto Chico Mendes de Conservação da Biodiversidade of the Ministry of Environment of Brazil (ICMBio) (MS-57450-1, 56912-1; MT-19838-6, 54728-1), which regulates wildlife sampling in Brazil. Biological samples were also reported to the National System for Access to Genetic Heritage and Associated Traditional Knowledge (SISGEN) according to the Law number 13.123/2015 and Decree 8772/2016. All wild mammals were captured and handled in accordance with guidelines of the American Society of Mammalogists for use of wild animals in research and recommendations for working with animals potentially infected with airborne pathogens [33,34]. Samplings of domestic animals on private property were authorized by the oral and written consent of owners.

## 3. Results

A total of 2068 vertebrates from 97 species were sampled, and 24,308 specimens of mosquitoes were collected and identified to at least 62 species (Table 1).

**Figure 1:**
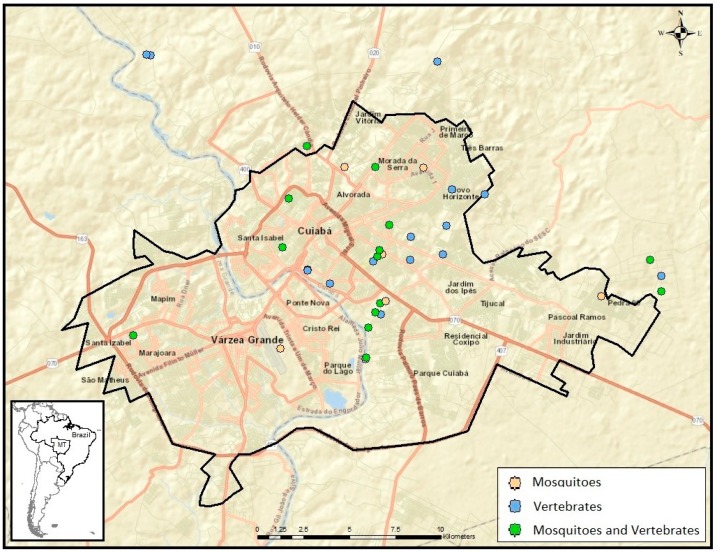
–Subsites used for sampling of mosquitoes and vertebrates in the metropolitan area of Mato Grosso state, West-Central region of Brazil.

**Figure 2:**
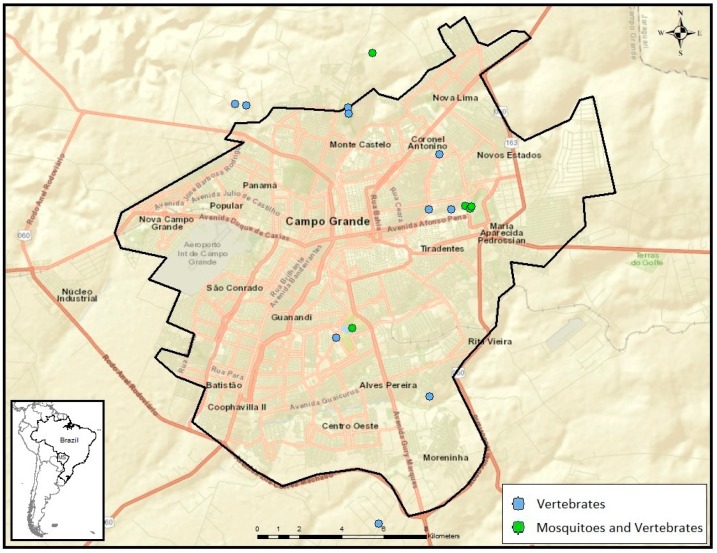
–Subsites used for sampling of mosquitoes and vertebrates in the metropolitan area of Mato Grosso do Sul state, West-Central region of Brazil.

Vertebrates and mosquitoes were collected in 46 sub-sites of MT, 36 being in the metropolitan area (Figure 1), and in 16 sub-sites of MS, 15 being in the metropolitan area (Figure 2). Domestic and wild vertebrates were captured in 35 subsites in MT and 16 subsites of MS, while mosquitoes were captured in 28 subsites in MT and five in MS.

In MT, 17 (37%) of 46 subsites had both vertebrate and mosquito samplings, 18 (39%) had only vertebrates sampled, and 11 (24%) had only mosquitoes sampled. In MS, five (31%) of 16 subsites had both mosquito and vertebrate samplings, and 11 (69%) had only vertebrates sampled. Vertebrate trapping effort included 16,065 m2-h of mist netting in MS and 11,668 m2-h in MT; 2850 Tomahawk trap-nights in MS and 1320 in MT; 1,890 Sherman trap-nights in MS and 720 in MT; and 150 turtle trap-nights in MS.

**Figure 3:**
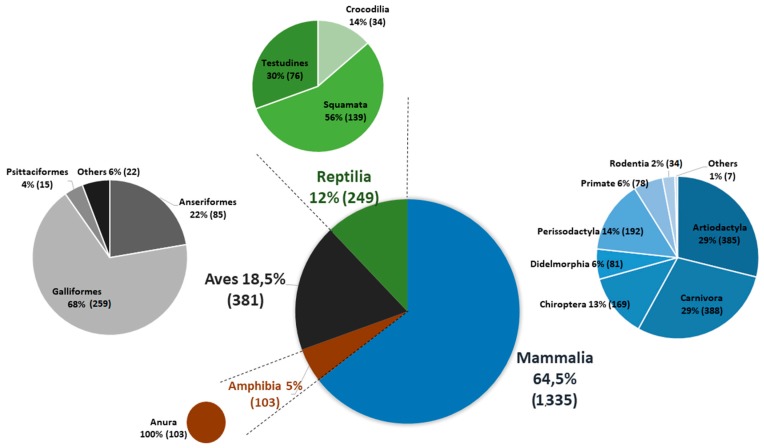
Schematic representing relative abundance of orders of domestic and wild vertebrates blood-sampled and tested for current and past infection with ZIKV.

Most abundant domestic species sampled were chicken (*n* = 226), dog (*n* = 190), horse (*n* = 189), cattle (*n* = 177), sheep (*n* = 170), and domestic goose (*n* = 63). Most common wildlife species were Cope’s toad (*Rhinella diptycha*, *n* = 92), South American coati (*Nasua nasua*, *n* = 86), white-eared opossum (*Didelphis albiventris*, *n* = 72), Geoffroy’s side-necked turtle (*Phyrnops geoffroanus*, *n* = 49), South American rattlesnake (*Crotalus durissus*, *n* = 48), and black-tailed marmoset (*Mico melanurus*, *n* = 48) (Figure 3).

Whole blood samples of 2064 animals were screened by real-time RT-PCR for the detection of acute flavivirus infections. Ninety-two (4.5%) presented a melting curve suggestive of flaviviral NS5 amplicons, but none of them confirmed positive for ZIKV either by specific real-time RT-PCR or nucleotide sequencing.

To investigate previous ZIKV exposure, plasma samples of 1498 animals of 62 species were tested by PRNT_90_ for the detection of ZIKV-neutralizing antibodies. From these, 34 (2.3%) from 11 species presented ZIKV-neutralizing antibodies (PRNT_90_ titer ≥20). Highest PRNT_90_ titers were observed in a flat-faced fruit-eating bat (*Artibeus planirostris*) presenting a titer of 2560, a dog (*Canis lupus familiaris*) with a titer of 1280, a white-cheeked spider monkey (*Ateles marginatus*) with a titer of ≥320, a crab-eating fox (*Cerdocyon thous*) with a titer of 160, and a hooded capuchin monkey (*Sapajus cay*) with a titer of 160 (Table 2). However, when these samples were tested by PRNT_90_ for other flaviviruses, including DENV-2, YFV, and WNV, aiming to discard potential heterologous reactions, only 23 animals of seven species showed a monotypic reaction and were considered seropositive for ZIKV. The animal that presented the highest monotypic PRNT_90_ titer for ZIKV was a white-cheeked spider monkey (*Ateles marginatus*) that presented a PRNT_90_ titer of 80 (Table 2).

**Figure 4:**
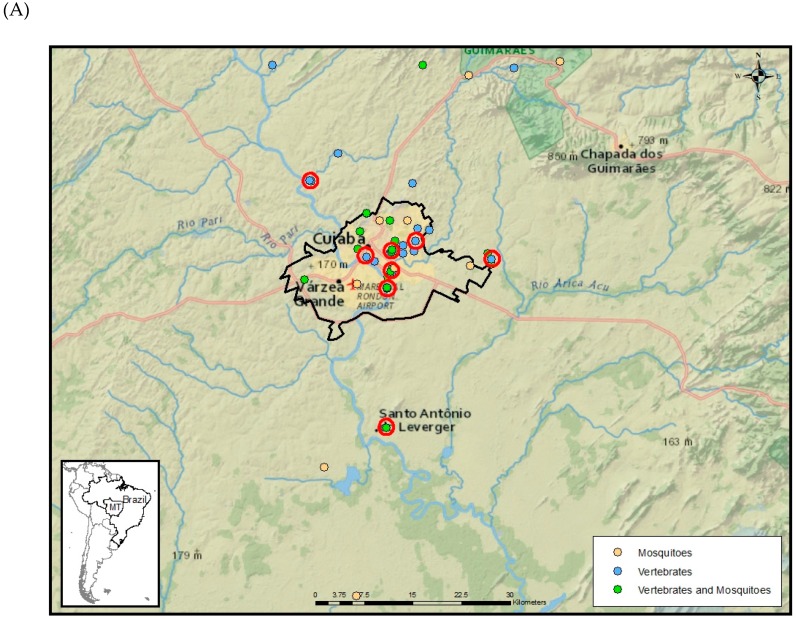

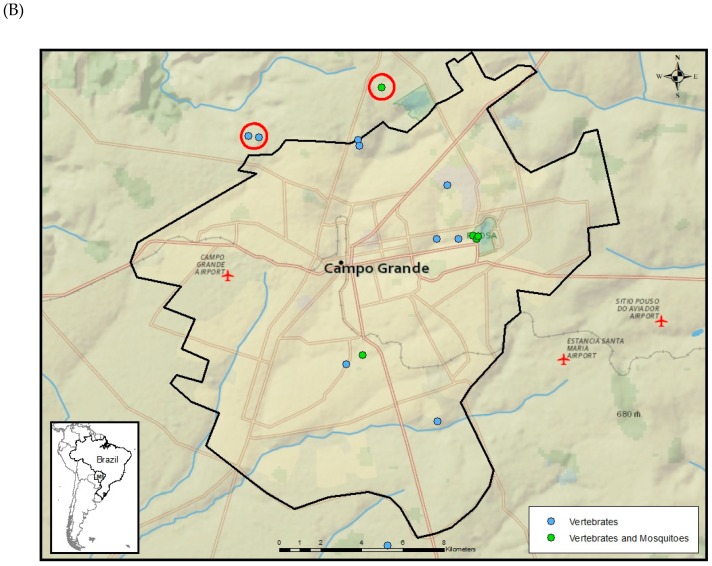
Subsites used for sampling of mosquitoes and vertebrates in Mato Grosso (**A**) and Mato Grosso do Sul states (**B**), West-Central region of Brazil. Red circles indicate subsites where animals presented monotypic antibody responses to ZIKV.

From the 23 animals that were seropositive for ZIKV, 17/639 (2.7%) were from MT and 6/859 (0.7%) from MS. In MT, eight (23%) of 35 subsites harbored animals with a monotypic reaction for ZIKV (Figure 4). In MS, two (12.5%) of the 16 subsites had seropositive animals for ZIKV (Figure 4).

Among the most abundant species tested that presented monotypic reactions for ZIKV, the highest positivity rate (seroprevalence) was observed in domestic graylag goose (10.8%), followed by cattle (2.9%), chickens (2.8%), horses (2.1%), dogs (1.9%), and sheep (1.2%) (Table 3).

A total of 24,308 adult mosquitoes were collected in MT and MS. Collected mosquitoes included engorged and non-engorged specimens, identified to at least 62 species. From these, 23,315 non-engorged mosquitoes of at least 60 species, being 16,091 (69%) males, 7223 (31%) females, and one that could not be sexed, were tested by real-time RT-PCR for flavivirus infection. Trapping efforts included 327 mosquito trap-nights and 15 h of aspiration in five subsites in MS, and 345 mosquito trap-nights and 75 h of aspiration in 28 subsites in MT.

From 23,315 specimens distributed in 1503 pools tested, 20,843 specimens (1263 pools) identified to at least 42 species were from 28 subsites of MT, and 2472 specimens (240 pools) of at least 47 species from five subsites of MS. From 1503 pools tested, 68 pools (4.5%) screened positive for flaviviruses, but all of these were negative for ZIKV by specific real-time RT-PCR. All 68 flavivirus-positive pools were submitted for nucleotide sequencing and 22 (32.4%) showed a certain degree of homology to a flavivirus, whereas the rest represented non-specific reactions. The majority of positive samples aligned closely to mosquito flavivirus, originally detected in *Aedes aegypti* from Madeira Island, Portugal, in 2010 (Genbank# HQ676625). Three samples showed similarity with the Kamiti river virus, an insect-specific flavivirus isolated in Kenya in 1999 [35]. One sample showed nonspecific low percent identity to the Geran virus, an orthonairovirus (Nairoviridae, Bunyavirales) isolated from ticks in Azerbaijan [36] (Table 4).

## 4. Discussion

Negative results from real-time RT-PCR in vertebrate and invertebrate species sampled in our study suggest no enzootic transmission of ZIKV between 2017 and 2018 in MT and MS. Local reports of ZIKV circulation in the neighboring human population during the same period of time [23,24] suggested epidemic rather than enzootic transmission of ZIKV in West-Central Brazil.

However, data presented here suggest that vertebrates have indeed been exposed to ZIKV in urban and peri-urban habitats. Among the seven species that generated a monotypic antibody response to ZIKV, six were domestic and only one was wild (Table 2). The only wild animal that was seropositive for ZIKV was kept in captivity in an urban area of Cuiabá. Among free-ranging wild species that showed ZIKV-neutralizing antibodies in heterotypic reactions were a flat-faced fruit-eating bat (*Artibeus planirostris*) and a crab-eating fox (*Cerdocyon thous*), species that can be found in peri-urban habitats in West-Central Brazil [37,38]. Monotypic reactions were detected in animals from different subsites mainly located in urban areas in MT and outside the metropolitan area of MS (Figure 4). Further analysis of the blood-engorged mosquitoes captured during this study, including the detection of vertebrate DNA in blood meals, might provide information regarding potential vector species involved in ZIKV spillover transmission to animals in West-Central Brazil.

According to our positivity criteria, ZIKV seroprevalence in non-human vertebrates was more than triple in MT (2.7%) compared to MS (0.7%) (Table 3). These results may reflect the higher incidence of human ZIKV cases reported in MT compared to MS, between 2017 and 2018. In 2017 and 2018, MT reported 63 and 16 cases per 100,000 residents, respectively. During the same years, MS reported 3 and 2.8 cases per 100,000 residents, respectively [39]. The percentage of subsites where animals presented monotypic ZIKV antibody responses was also unequal, being 23% of the subsites sampled with monotypic reactions for ZIKV in MT, and 12.5% in MS (Figure 4).

One of the strengths of our study is the broad sampling of potential hosts and vectors for ZIKV in metropolitan areas of two large cities in West-Central Brazil. A total of 2,068 vertebrates of 97 species, including 81 wild and 16 domestic species from 16 different locations in MS and 34 locations in MT, were tested. Among wild species, over 170 Chiroptera (bats), 100 Anura (toads), 80 primates (monkeys and marmosets), and 80 Didelphimorphia (opossums) were molecularly tested for active ZIKV infection. Neutralizing antibodies for ZIKV were evaluated by PRNT_90_ in 62 species, including 49 wild species and 13 domestic species. Of the 49 wild species tested, 35 (73%) corresponded to captured free-ranging individuals. From the total of 523 wild animals tested, 459 (88%) were free-ranging.

Among mosquitoes, roughly 23,300 specimens captured in more than 30 subsites and identified to around 60 species were tested, including over 20,000 *Culex* spp., 700 *Psorophora* spp., 440 *Aedes aegypti*, 340 *Anopheles* spp., and 300 *Aedes albopictus*. However, few *Haemagogus*- and *Sabathes*-genus mosquitoes were collected in our field sites. These genera have been implicated as zoonotic vectors for the yellow fever virus in Brazil [40].

Another strength is the usage of both highly sensitive and highly specific molecular and serological methods with conservative criteria of positivity for detection of ZIKV infection. Molecular methods included two real-time RT-PCR protocols (SYBR and TaqMan) followed by Sanger nucleotide sequencing. Only samples confirmed by nucleotide sequencing were considered positive. Antibody detection in plasma was accomplished by PRNT_90_, which is considered the most specific serological test for the differentiation of flavivirus infections in convalescent serum samples [41,42]. The PRNT utilized a highly conservative threshold of 90% neutralization, and only samples that tested positive for ZIKV but negative for WNV, DENV-2, and YFV, were confirmed seropositive for ZIKV.

Limitations of our study include the limited number of flaviviruses used in the differential diagnosis. Despite using DENV-2, which is the most prevalent serotype of DENV in West-Central Brazil, and YFV that is the most common enzootic flavivirus in Brazil, additionally only WNV was used to minimize the detection of cross-reactive samples. Although we only considered as ZIKV-seropositive those monotypic reactions with no indication of cross-reaction among three flaviviruses, there may be other active flaviviruses circulating in our study sites. A dozen flaviviruses of medical importance have been reported in Brazil, including several zoonotic viruses such as Ilheus, Rocio, and St. Louis encephalitis viruses. Cross-reactivity to other flaviviruses could not be fully discarded.

Another limitation of our study was the irregular number of primate samples per study site. From 78 primates tested, 58 (74%), of five species, were mostly free-ranging from five subsites of MT, and 20 (26%), of four species kept in captivity, were from a single wild animal rescue center of MS. Thus, the exposure of non-human primates to ZIKV in MS may be underestimated.

Mosquito testing presented another limitation. From 23,315 non-engorged mosquitoes tested, 16,091 (69%) were males. Male mosquitoes are not hematophagous and were only included in the present study to identify potential vertical or venereal transmission of medically important arboviruses. Surprisingly though, from 24 flavivirus-positive pools, 17 (71%) were from females, and only seven (29%) were from males. Although no mosquitoes were positive for human–pathogenic arboviruses, insect-specific viruses were detected in six genera. Similar results were observed in a mosquito arbovirus survey conducted in Kenya in 2012 [43].

It is noteworthy that from 34 animals that presented neutralizing antibodies for ZIKV, only 23 were considered seropositive for ZIKV. The remaining 11 animals, including animals presenting high titers like a flat-faced fruit-eating bat with a PRNT_90_ titer of 2560, were not considered seropositive for ZIKV for being reactive not only to ZIKV but also to another flavivirus. Thus, animals that were exposed not only to ZIKV, but also to another flavivirus were considered as presenting a heterotypic reaction, and thus, the number of animals exposed to ZIKV might be underestimated.

## 5. Conclusions

Although we cannot exclude the potential circulation of ZIKV in non-human vertebrates and wild mosquitoes in Brazil, according to our results, no active circulation of ZIKV was detected in potential wild and domestic vertebrate hosts between May 2017 and March 2018 in West-Central Brazil. The detection of neutralizing antibodies in monotypic reactions to ZIKV in roughly 30 animals of mostly domestic species, suggests that vertebrates have indeed been exposed to ZIKV in urban and peri-urban habitats of West-Central Brazil.

## Figures and Tables

**Table 1:** Vertebrate and mosquito species sampled in West-Central Brazil between 2017 and 2018 with a sample size of N > 15 (vertebrates) and N > 20 (mosquitoes), broken down by state. MT, state of Mato Grosso; MS, state of Mato Grosso do Sul.

	Total	MT	MS		Total	MT	MS
Vertebrate Species	N	N (%)	N (%)	Mosquito Species	N	N (%)	N (%)
*Gallus gallus domesticus*	226	154 (68)	72 (32)	*Culex* spp.	21,207	19,215 (91)	1992 (9)
*Canis lupus familiaris*	190	79 (42)	111 (58)	*Aedes (Stegomyia) aegypti*	479	430 (90)	49 (10)
*Equus ferus caballus*	189	90 (48)	99 (52)	*Aedes (Stegomyia) albopictus*	343	311 (91)	32 (9)
*Bos indicus/taurus*	177	64 (36)	113 (64)	*Culex (Culex) quinquefasciatus*	222	222 (100)	0 (0)
*Ovis aries*	170	53 (31)	117 (69)	*Psorophora (Grabhamia) dimidiata*	197	83 (42)	114 (58)
*Felis silvestris catus*	102	52 (51)	50 (49)	*Culex (Culex) nigripalpis*	175	175 (100)	0 (0)
*Rhinella diptycha*	92	57 (62)	35 (38)	*Wyeomyia* spp.	167	167 (100)	0 (0)
*Nasua nasua*	86	4 (5)	82 (95)	*Aedes (Ochlerotatus) scapularis*	148	55 (37)	93 (63)
*Didelphis albiventris*	72	56 (78)	16 (22)	*Anopheles* spp.	143	77 (54)	66 (46)
*Anser anser domesticus*	63	63 (100)	0 (0)	*Psorophora (Janthinosoma) albigenu*	139	139 (100)	0 (0)
*Phrynops geoffroanus*	49	4 (8)	45 (92)	*Psorophora (Janthinosoma) ferox*	114	114 (100)	0 (0)
*Mico melanurus*	48	48 (100)	0 (0)	*Psorophora* spp.	69	39 (56,5)	30 (43,5)
*Crotalus durissus*	48	0 (0)	48 (100)	*Aedes* spp.	65	26 (40)	39 (60)
*Artibeus lituratus*	38	19 (50)	19 (50)	*Anopheles (Nyssorhynchus) rangeli*	64	64 (100)	0 (0)
*Artibeus planirostris*	38	5 (13)	33 (87)	*Deinocerites* spp.	64	64 (100)	0 (0)
*Sus scrofa domesticus*	36	36 (100)	0 (0)	*Limatus* spp.	58	58 (100)	0 (0)
*Carollia perspicillata*	36	21 (58)	15 (42)	*Anopheles (Stethomyia) kompi*	55	0 (0)	55 (100)
*Bothrops moojeni*	36	0 (0)	36 (100)	*Haemagogus (Haemagogus) janthinomys*	50	0 (0)	50 (100)
*Caiman yacare*	34	34 (100)	0 (0)	*Mansonia* spp.	49	49 (100)	0 (0)
*Bothrops alternatus*	24	0 (0)	24 (100)	*Psorophora (Psorophora) cilipes*	37	0 (0)	37 (100)
*Hydrochoerus hydrochaeris*	23	1 (4)	22 (96)	*Psorophora (Grabhamia) cingulata*	35	0 (0)	35 (100)
*Phasianus colchicus*	17	17 (100)	0 (0)	*Uranotaenia* spp.	30	30 (100)	0 (0)
				*Anopheles (Nyssorhynchus) benarrochi*	29	0 (0)	29 (100)

**Table 2:** Plasma samples of domestic and wild animals from West-Central Brazil with neutralizing antibody titers for ZIKV (PRNT_90_ titer ≥20). Titer determined by 90% plaque–reduction neutralization test. ZIKV = Zika virus; YFV = yellow fever virus; DENV-2 = dengue 2 virus; WNV = West Nile virus; ID = sample identifier; MS = Mato Grosso do Sul; MT = Mato Grosso.

Class	Order	Species	ID	State	ZIKV	YFV	DENV-2	WNV	Monotypic
Mammalia	Primates	*Ateles marginatus*	AU0002	MT	80	<10	<10	<10	YES
Mammalia	Perissodactyla	*Equus ferus caballus*	AU0194	MT	40	<10	<10	<10	YES
Mammalia	Artiodactyla	*Ovis aries*	AU0199	MT	40	<10	<10	<10	YES
Mammalia	Artiodactyla	*Bos indicus/taurus*	AU0219	MT	20	<10	<10	<10	YES
Mammalia	Artiodactyla	*Bos indicus/taurus*	AU0274	MT	20	<10	<10	<10	YES
Mammalia	Carnivora	*Canis lupus familiaris*	AU0063	MT	20	<10	<10	<10	YES
Mammalia	Perissodactyla	*Equus ferus caballus*	AU0137	MT	20	<10	<10	<10	YES
Mammalia	Perissodactyla	*Equus ferus caballus*	AU0188	MT	20	<10	<10	<10	YES
Mammalia	Perissodactyla	*Equus ferus caballus*	AU0192	MT	20	<10	<10	<10	YES
Mammalia	Artiodactyla	*Ovis aries*	AU0205	MT	20	<10	<10	<10	YES
Aves	Anseriformes	*Anser anser domesticus*	AU0439	MT	20	<10	<10	<10	YES
Aves	Anseriformes	*Anser anser domesticus*	AU0440	MT	20	<10	<10	<10	YES
Aves	Anseriformes	*Anser anser domesticus*	AU0445	MT	20	<10	<10	<10	YES
Aves	Anseriformes	*Anser anser domesticus*	AU0447	MT	20	<10	<10	<10	YES
Aves	Galliformes	*Gallus gallus domesticus*	AU0008	MT	20	<10	<10	<10	YES
Aves	Galliformes	*Gallus gallus domesticus*	AU0029	MT	20	<10	<10	<10	YES
Aves	Galliformes	*Gallus gallus domesticus*	AU0090	MT	20	<10	<10	<10	YES
Mammalia	Artiodactyla	*Bos indicus/taurus*	AG0329	MS	40	<10	<10	<10	YES
Mammalia	Artiodactyla	*Bos indicus/taurus*	AG0340	MS	20	<10	<10	<10	YES
Mammalia	Artiodactyla	*Bos indicus/taurus*	AG0348	MS	20	<10	<10	<10	YES
Mammalia	Carnivora	*Canis lupus familiaris*	AG0007	MS	20	<10	<10	<10	YES
Mammalia	Carnivora	*Canis lupus familiaris*	AG0019	MS	20	<10	<10	<10	YES
Aves	Galliformes	*Gallus gallus domesticus*	AG0379	MS	20	<10	<10	<10	YES
Mammalia	Artiodactyla	*Bos indicus/taurus*	AG0346	MS	20	≥10	≥10	<10	NO
Mammalia	Primates	*Sapajus cay*	AG0297	MS	160	<10	≥10	<10	NO
Mammalia	Perissodactyla	*Equus ferus caballus*	AU0366	MT	40	≥10	≥10	≥10	NO
Mammalia	Rodentia	*Dasyprocta azarae*	AU0500	MT	80	<10	≥10	<10	NO
Mammalia	Perissodactyla	*Equus ferus caballus*	AU0184	MT	80	<10	<10	≥10	NO
Mammalia	Carnivora	*Cerdocyon thous*	AU0197	MT	160	<10	≥10	<10	NO
Mammalia	Carnivora	*Canis lupus familiaris*	AU0257	MT	1280	<10	<10	≥10	NO
Mammalia	Chiroptera	*Artibeus planirostris*	AEU042	MT	2560	<10	≥10	≥10	NO
Mammalia	Primates	*Ateles marginatus*	AU0001	MT	≥320	<10	≥10	<10	NO
Aves	Anseriformes	*Anser anser domesticus*	AU0438	MT	20	<10	≥10	<10	NO
Aves	Galliformes	*Gallus gallus domesticus*	AU0375	MT	80	<10	<10	≥10	NO

**Table 3:** Seroprevalence based upon monotypic responses to ZIKV among species with a sample size of N >12, broken down by state, West-Central Brazil. MT, state of Mato Grosso; MS, state of Mato Grosso do Sul.

Species		Total		MT		MS	
Scientific Name	Common Name	N	M (%)	N	M (%)	N	M (%)
*Anser anser domesticus*	Domestic graylag goose	37	4 (10.8)	37	4 (10.8)	0	_
*Bos indicus/taurus*	Cattle	171	5 (2.9)	59	2 (3.4)	112	3 (2.7)
*Gallus gallus domesticus*	Chicken	145	4 (2.8)	102	3 (2.9)	43	1 (2.3)
*Equus ferus caballus*	Horse	187	4 (2.1)	88	4 (4.5)	99	0
*Canis lupus familiaris*	Dog	159	3 (1.9)	48	1 (2.1)	111	2 (1.8)
*Ovis aries*	Sheep	164	2 (1.2)	49	2 (4.1)	115	4 (3.5)
*Nasua nasua*	South American coati	82	0	2	0	80	0
*Felis catus*	Cat	66	0	23	0	43	0
*Phrynops geoffroanus*	Geoffroy’s side-necked turtle	41	0	0	_	41	0
*Didelphis albiventris*	White-eared opossum	38	0	27	0	11	0
*Artibeus lituratus*	Great fruit-eating bat	32	0	16	0	16	0
*Artibeus planirostris*	Flat-faced fruit-eating bat	32	0	5	0	27	0
*Crotalus durissus*	South American rattlesnake	32	0	0	_	32	0
*Sus scrofa domesticus*	Pig	32	0	32	0	0	_
*Mico melanurus*	Black-tailed marmoset	31	0	31	0	0	_
*Rhinella diptycha*	Cope’s toad	26	0	13	0	13	0
*Carollia perspicillata*	Seba’s short-tailed bat	23	0	11	0	12	0
*Bothrops moojeni*	Moojen’s lancehed	22	0	0	_	22	0
*Caiman yacare*	Yacare caiman	22	0	22	0	0	_
*Hydrochoerus hydrochaeris*	Capybara	21	0	0	_	21	0
*Bothrops alternatus*	Urutu lancehead	15	0	0	_	15	0
*Sapajus cay*	Hooded capuchin	12	0	0	_	12	0
All other species *	General	108	0	74	0	34	0

* Other species include: *Alouatta caraya*, *Anas platyrhynchos domesticus*, *Ateles marginatus*, *Anser cygnoides*, *Aotus lemurinus*, *Artibeus fimbriatus*, *Bothrops mattogrossensis*, *Cairina moschata*, *Callithrix jacchus*, *Callithrix penicillata*, *Capra aegagrus hircus*, *Carollia benkeithi*, *Cerdocyon thous*, *Chiroderma trinitatum*, *Dasyprocta azarae*, *Didelphis aurita*, *Didelphis marsupialis*, *Iguana iguana*, *Leptodactylus labyrinthicus*, *Meleagris gallopavo*, *Molossops temminckii*, *Molossus molossus*, *Molossus rufus*, *Myotis nigricans*, *Myrmecophaga tridactyla*, *Noctilio albiventris*, *Phasianus colchicus*, *Phylloderma stenops*, *Platyrrhinus lineatus*, *Podocnemis expansa*, *Puma concolor*, *Rhea americana*, *Salvator merianae*, *Sapajus apella*, *Tapirus terrestres*, *Trachemys dorbigni*, *Trachemys scripta*, *Tupinambis teguixin*, unidentified Rodentia.

**Table 4:** Mosquito pools from the West-Central region of Brazil that tested positive by flavivirus real-time RT-PCR and nucleotide sequences aligned with known viruses in GenBank.

Pool ID	State	Species	n	Coverage/Identity	Virus Sequences	Genbank#
P0048	MS	*Sabethes* sp.	1	34%/95.7%	Mosquito flavivirus	HQ676625.1
P0049	MS	*Sabethes* sp.	1	84%/92.2%	Mosquito flavivirus	HQ676625.1
P0057	MS	*Aedes (Stegomyia) aegypti*	2	50%/87.4%	Mosquito flavivirus	HQ676625.1
P0217	MS	*Aedes (Stegomyia) aegypti*	4	78%/84.5%	Mosquito flavivirus	HQ676625.1
PU0026	MT	*Aedes* sp.	3	77%/95.0%	Mosquito flavivirus	HQ676624.1
PU0064	MT	*Culex* sp.	12	46%/80.0%	Kamiti river virus	AY149904.1
PU0196	MT	*Culex* sp.	25	87%/92.0%	Mosquito flavivirus	HQ676625.1
PU0259	MT	*Limatus* sp.	23	77%/93.0%	Mosquito flavivirus	HQ676625.1
PU0261	MT	*Limatus* sp.	25	86%/90.0%	Mosquito flavivirus	HQ676625.1
PU0330	MT	*Wyeomyia* sp.	25	87%/93.9%	Mosquito flavivirus	HQ676625.1
PU0806	MT	*Aedes (Stegomyia) aegypti*	18	54%/78.3%	Kamiti river virus	DQ335465.1
PU0807	MT	*Aedes (Stegomyia) aegypti*	25	77%/89.2%	Mosquito flavivirus	HQ676625.1
PU0808	MT	*Aedes (Stegomyia) aegypti*	25	78%/91.4%	Mosquito flavivirus	HQ676625.1
PU0852	MT	*Aedes* sp.	2	78%/86.8%	Mosquito flavivirus	HQ676625.1
PU0853	MT	*Aedes* sp.	2	80%/85.9%	Mosquito flavivirus	HQ676625.1
PU0874	MT	*Psorophora (Janthinosoma) albigenu*	25	37%/92.6%	Geran virus	KP792714.1
PU1001	MT	*Aedes (Stegomyia) aegypti*	1	94%/94.7%	Mosquito flavivirus	HQ676625.1
PU1007	MT	*Aedes (Stegomyia) aegypti*	2	80%/89.2%	Mosquito flavivirus	HQ676625.1
PU1031	MT	*Aedes (Stegomyia) aegypti*	2	53%/80.8%	Kamiti river virus	DQ335465.1
PU1032	MT	*Aedes (Stegomyia) aegypti*	15	82%/87.6%	Mosquito flavivirus	HQ676625.1
PU1098	MT	*Aedes (Stegomyia) aegypti*	7	82%/87.6%	Mosquito flavivirus	HQ676625.1
PU1124	MT	*Aedes (Stegomyia) aegypti*	6	86%/94.7%	Mosquito flavivirus	HQ676625.1
